# Metabolic Syndrome features and risk of neural tube defects

**DOI:** 10.1186/1471-2393-7-21

**Published:** 2007-09-19

**Authors:** Joel G Ray, Miles D Thompson, Marian J Vermeulen, Chris Meier, Philip R Wyatt, Pui-Yuen Wong, Anne M Summers, Sandra A Farrell, David EC Cole

**Affiliations:** 1 Departments of Medicine, Obstetrics and Gynecology and Health Policy Management and Evaluation, St. Michael's Hospital, University of Toronto, 30 Bond Street, Toronto, Ontario, M5B 1W8, Canada; 2 Department of Clinical Biochemistry, University of Toronto, Toronto, Ontario, Canada; 3 Institute for Clinical Evaluative Sciences, University of Toronto, Toronto, Ontario, Canada; 4 Department of Genetics, North York General Hospital, Toronto, Ontario, Canada; 5 Department of Genetics, York Central Hospital, Richmond Hill, Ontario, Canada; 6 Regional Genetics Program, The Credit Valley Hospital, Mississauga, Ontario, Canada; 7 Department of Laboratory Medicine, Sunnybrook Health Sciences Centre, University of Toronto, Toronto, Ontario, Canada

## Abstract

**Background:**

Maternal obesity and pre-pregnancy diabetes mellitus, features of the metabolic syndrome (MetSyn), are individual risk factors for neural tube defects (NTD). Whether they, in combination with additional features of MetSyn, alter this risk is not known. We evaluated the risk of NTD in association with maternal features of the MetSyn.

**Methods:**

We used a population-based case-control study design in the province of Ontario, Canada. Cases and controls were derived from women who underwent antenatal maternal screening (MSS) at 15 to 20 weeks' gestation. There were 89 maternal cases with, and 434 controls without, an NTD-affected singleton pregnancy. Maternal features of MetSyn were defined by the presence of pre-pregnancy diabetes mellitus, body weight ≥ 90th centile among controls, non-white ethnicity and/or serum highly sensitive C-reactive protein (hsCRP) ≥ 75th centile of controls. Since hsCRP naturally increases in pregnancy, analyses were performed with, and without, the inclusion of hsCRP in the model.

**Results:**

Mean hsCRP concentrations were exceptionally high among study cases and controls (6.1 and 6.4 mg/L, respectively). When hsCRP was excluded from the model, the adjusted odds ratios for NTD were 1.9 (95% confidence interval 1.1–3.4) in the presence 1 feature of MetSyn, and 6.1 (1.1–32.9) in the presence of 2 or more features. When hsCRP was included, the respective risk estimates were attenuated to 1.6 (0.88–2.8) and 3.1 (1.2–8.3).

**Conclusion:**

We found about 2-fold and 6-fold higher risk for NTD in the presence 1, and 2 or more features, of the metabolic syndrome, respectively. It is not clear whether this risk is altered by the presence of a high serum hsCRP concentration.

## Background

The risk of neural tube defects (NTD) – spina bifida and anencephaly – is significantly reduced by a periconceptional intake of folic acid [1]. While a large proportion of NTD may be preventable, risk factors for NTD that are independent of folate metabolism, including maternal obesity [2] and pre-pregnancy diabetes mellitus (DM) may persist [3].

The metabolic syndrome (MetSyn) is characterized by a cluster of metabolic risk factors including abdominal obesity, DM or insulin resistance, non-white ethnicity [4], dyslipidemia, chronic hypertension and elevated inflammatory highly sensitive serum C-reactive protein (hsCRP) [5]. Since 40% of Western teenagers exhibit a sedentary lifestyle [6] and 5–16% are obese [6,7], MetSyn is now seen in 15% of women who are in their reproductive years [8].

If obesity and pre-pregnancy DM are each independent risk factors for NTD, then it is valuable to know how they interact together, and with other features of MetSyn, namely, non-white ethnicity and elevated serum hsCRP. We examined whether these selected features of MetSyn are additively associated with an increased risk of NTD.

## Methods

We performed a population-based case-control study. Details of the study methods are described elsewhere [9,10]. Briefly, since 1993, standardized maternal serum screening (MSS) was made available to all women at 15 to 20 weeks' gestation through a physician or midwife, with a mean rate of use of more than 60%. The current study comprised the period from 1994 to late 2000. Self-declared maternal date of birth, gravidity, ethnicity, current weight, and the presence of prepregnancy DM were recorded in a standardized fashion on the MSS Requisition Sheet, completed by the caregiver at the time of screening. Open NTD cases were detected antenatally by ultrasonography or fetal autopsy, or postnatally through data linkage of the mother's Ontario health insurance number with that of her infant during the delivery hospitalization, through the Canadian Institute for Health Information Discharge Abstract Database [9,10].

For each case, five maternal controls without an NTD-affected pregnancy were randomly selected within a +/- 24-month period. Serum samples, originally collected at the time of MSS in the index singleton pregnancy, were frozen at -70°C. Each frozen sample was thawed and analyzed for hsCRP using the DPC Immulite 2000 immunoassay analyzer (Diagnostic Products Corporation, Los Angeles, CA), whose functional sensitivity is 0.2 mg/L, with within- and between-run CVs of 2.8 % and 3.1 % at 3.2 mg/L, and 3.4 % and 3.8 % at 21.8 mg/L, respectively. Serum folate was analyzed using the Bayer Centaur immunoassay analyzer method (Bayer Diagnostics Division, Toronto, ON). The distribution of serum hsCRP and folate values was positively skewed, so we log-transformed these measures and used inverse transformations to generate geometric means and standard deviations (SD).

### Statistical analysis

Maternal characteristics were compared between groups using a non-paired t-test for continuous variables, a median or Wilcoxon rank-sum test for non-parametric data, and the chi-square test or Fisher's exact test for categorical data.

Maternal MetSyn features were defined as follows: 1) presence of pre-pregnancy DM; 2) weight ≥ 90th centile value of the controls; 3) non-white ethnicity; and 4) serum hsCRP ≥ 75th centile value in controls.

Crude and adjusted odds ratios (OR) and 95% confidence intervals (CI) were derived using unconditional logistic regression analysis, comparing women with 0, 1 or ≥ 2 features of MetSyn, with 0 features as the referent. We adjusted for maternal age (1-year increments) and serum folate concentration at the time of MSS, as well as the prevalence of low-income status within each participant's neighborhood. The latter was defined as less than the low-income cut-off for all Ontarians [10]. The forward sortation area of each participant (i.e., the first three characters of her postal code) was used to identify each woman's neighborhood, from the 2001 Canadian Census. The trend in the risk of NTD according to the number of features of the MetSyn was evaluated using a Cochran-Armitage test for trend.

Since hsCRP increases in pregnancy, distinguishing an abnormal from a physiologically elevated value is difficult [11], so the above modeling was performed with and without the inclusion of hsCRP.

Means and SD were computed and compared using an unpaired t-test. All variables were included in the model a priori, and statistical significance was set at a 2-sided p < 0.05. Analyses were performed using SAS Version 8.0 (SAS Institute, Cary, NC). The study was done in accordance with a research protocol approved through the Ministry of Health and Longterm Care in Ontario, and approval of the Research Ethics Boards of St. Michael's Hospital and The North York General Hospital, with all participant identifiers removed prior to analysis.

## Results

We included 89 NTD-affected pregnancies and 434 unaffected pregnant controls (Table 1). All cases had a minimum of four controls available, and the majority had five. About half (263) of all participants were from the pre-folic acid fortification era and 260 were from the period after. Of all 89 NTD cases, 67 (75.3%) were diagnosed antenatally.

**Table 1:** Participant characteristics

**Maternal characteristic**	**Cases of women with an NTD-affected pregnancy (n = 89)**	**Controls of women without an NTD-affected pregnancy (n = 434)**	**P-value, comparing cases and controls**
Mean (SD) age, years	28.6 (4.8)	29.8 (5.1)	0.04
Median gravidity	2.0	2.0	0.02
Weight, kg			
*Mean (SD)*	68.6 (15.5)	67.4 (13.5)	0.50
*90th centile*	87.6	83.9*	0.50
No. (%) with prepregnancy diabetes mellitus	3 (3.4)	5 (1.2)	0.14
Ethnicity (No. [%])			
*White*	56 (70.9)	367 (85.8)	0.01
*Asian*	16 (20.2)	41 (9.6)	
*Black*	5 (6.3)	12 (2.8)	
*Other*	2 (2.5)	8 (1.9)	
Mean (SD) % with low income status in the local population	12.9 (6.7)	13.2 (7.5)	0.77
Geometric mean (SD) serum folate, nmol/L	13.3 (3.0)	13.9 (2.8)	0.96
Serum highly sensitive C-reactive protein, mg/L			
*Geometric mean (SD)*	6.1 (2.9)	6.4 (2.3)	0.92
*75th centile*	13.0	11.5*	--

*Used to define the abnormal cut-point in the current study.

Mean serum hsCRP were 6.1 and 6.4 mg/L in the cases and controls, respectively (Table 1). When hsCRP was excluded from the model, the crude OR for NTD were 1.9 (1.2–3.1) and 6.1 (1.2–30.9) in the presence 1 and 2 or more features of MetSyn, respectively, relative to none (trend p = 0.001) (Table 2). The corresponding adjusted OR were essentially the same (Table 2). With hsCRP included as a feature of MetSyn, the adjusted OR for NTD was 1.6 (0.88–2.8) in the presence of 1 feature, and 3.1 (1.2–8.3) in the presence of at least 2 features, relative to none (trend p = 0.009) (Table 2).

**Table 2:** Risk of open neural tube defects in relation to certain maternal features of the metabolic syndrome (MetSyn)

		**Prevalence of MetSyn**		**Odds ratio (95% CI)**		
**Model**	**No. features of MetSyn**	**No. (%) cases**	**No. (%) controls**	**Crude**	Adjusted**	**P-value for trend**

***Without hsCRP as a feature of MetSyn***	0 (n = 382)	54 (60.7)	328 (75.6)	1.0 (ref)	1.0 (ref)	0.001
	1 (n = 135)	32 (36.0)	103 (23.7)	1.9 (1.2–3.1)	1.9 (1.1–3.4)	
	≥ 2 (n = 6)	3 (3.4)	3 (0.69)	6.1 (1.2–30.9)	6.1 (1.1–32.9)	
						
With hsCRP as a feature of MetSyn*	0 (n = 351)	50 (56.2)	301 (69.4)	1.0 (ref)	1.0 (ref)	0.009
	1 (n = 147)	32 (36.0)	115 (26.5)	1.7 (1.0–2.7)	1.6 (0.88–2.8)	
	≥ 2 (n = 25)	7 (7.8)	18 (4.1)	2.3 (0.93–5.9)	3.1 (1.2–8.3)	

*Not all participants had a serum sample available for hsCRP evaluation

**Adjusted for maternal age (1-year increments), serum folate concentration and low income status.

hsCRP highly-sensitive C-reactive protein; CI confidence interval

In a *post hoc *analysis that excluded both hsCRP and ethnicity from the models, the crude OR were 1.5 (0.84–2.7) and 1.4 (0.70–2.8) for 1 and 2 or more features of the MetSyn, respectively. The corresponding adjusted OR were 1.4 (0.70–2.8) and 1.5 (0.40–5.7).

## Discussion

In the presence of at least one feature of MetSyn in pregnancy, the risk of NTD was nearly doubled. In the presence of two or more features, it was six times higher. Inclusion of hsCRP as a MetSyn feature attenuated these risk estimates considerably.

Our study was limited by the manner in which the features of MetSyn were determined. Its retrospective design enabled us to assess only a sub-set of these features, and neither chronic hypertension nor dyslipidemia [5] were included herein. While we evaluated self-reported maternal weight, this was not paired with height, nor was it measured before pregnancy. However, there appears to be little variation between self-reported and measured weight in pregnancy [12,13]. Furthermore, maternal weight and body mass index (BMI) are highly correlated [14,15], as are second trimester and pre-pregnancy BMI [16]. Our inclusion of women with pre-pregnancy DM did not distinguish between those with type 1 and type 2 DM, nor did our use of broad ethnic categories enable us to account for the additional within-group diversity, including country of birth. Nonetheless, others have emphasized the utility of "non-white" ethnicity as a determinant of risk for MetSyn [8,17,18]. We previously observed that women of First Nations ancestry had about a five times higher risk of NTD compared to White women, which was not so among women of other non-White ethnic origins [19]. Finally, it was not known how many cases or controls were taking folic acid tablet supplements periconceptionally, a major determinant of NTD risk [1], although we did adjust for serum folate status at the time of screening, as well as socioeconomic status, which itself is a predictor of periconceptional folic acid supplement use [20].

Including hsCRP as a feature of MetSyn attenuated the risk of NTD in this study. A likely explanation is the physiological effect of pregnancy on hsCRP [11]. This was reflected by an exceptionally high mean hsCRP concentrations among cases and controls [21], which likely diminished our ability to detect any association, if truly present. Recent evidence from the Framingham Offspring Study suggests that combining hsCRP in a model of MetSyn does not improve the ability to predict cardiovascular disease risk [21]. Clearly, measuring hsCRP prior to conception, or months after completion of pregnancy, would better a better test of its utility for predicting NTD risk.

Which features of MetSyn best predict the risk of NTD? Shaw and colleagues found that both a high-caloric diet [22] and a sedentary lifestyle [23] were independent risk factors for NTD. In their Californian population-based case-control study, the risk of NTD was highest among women who mean dietary glycemic index was in the upper vs. lower quartile (adjusted OR 1.9, 95% CI 1.3–2.7), especially when periconceptional BMI was also elevated (adjusted OR 4.0, 95% CI 1.0–15.7) [22]. Other studies, including ours, have confirmed the likely role of maternal obesity and DM as risk factors for NTD [2,3]. While it may be worthwhile to include both hypertriglyceridemia and chronic hypertension in a MetSyn model of NTD risk, they too are better measured in the non-pregnant state, as they are altered by pregnancy [24]. Chronic hypertension could conceivably alter the risk of NTD based on the link to maternal hyperhomocysteinemia [25], but it is possible that there are other biologically plausible explanations.

Fewer than 10% of maternal cases studied herein exhibited two or more features of MetSyn. Nonetheless, because MetSyn is now so prevalent among adults and children [7,8], this figure likely has importance at both the individual and societal levels. Most features of MetSyn can be inexpensively determined within an outpatient clinical setting, including measurement of BMI or pre-pregnancy waist circumference, blood pressure and capillary blood glucose concentration. We suggest that women considering pregnancy who are at risk for MetSyn be advised to increase dietary and supplemental intakes of folic acid, and consider a path of healthy eating and regular physical activity. More evidence is needed to determine whether maternal "metabolic modification" [26] can safely and efficaciously lower the risk of NTD.

## Conclusion

These preliminary findings suggest that there is a higher associated risk of NTD in the presence of features of the MetSyn. It is not clear whether this risk is altered by the additional presence of a high serum hsCRP concentration.

## Competing interests

The author(s) declare that they have no competing interests.

## Authors' contributions

JGR and MDT conceived of the study. MDT, CM, PRW, PYW, AMS, SAF and DEC coordinated the study. JGR, MJV, CM and PYW carried out the data analysis. MJV carried out the statistical analysis. JGR, MJV, PRW and DEC wrote the manuscript. All authors read and approved the final manuscript.

## Pre-publication history

The pre-publication history for this paper can be accessed here:


